# Cellular activation status in femoral shaft fracture hematoma following different reaming techniques – A large animal model

**DOI:** 10.1002/jor.25309

**Published:** 2022-03-17

**Authors:** Michel Paul Johan Teuben, Sascha Halvachizadeh, Yannik Kalbas, Zhi Qiao, Nikola Cesarovic, Miriam Weisskopf, Henrik Teuber, Miriam Kalbitz, Paolo Cinelli, Roman Pfeifer, Hans‐Christoph Pape, TREAT Research Group

**Affiliations:** ^1^ Department of Traumatology University Hospital Zurich Zurich Switzerland; ^2^ Harald Tscherne Laboratory for Orthopedic Research Zurich Switzerland; ^3^ Department of Trauma and Reconstructive Surgery University Clinic RWTH Aachen Aachen Germany; ^4^ Division of Surgical Research University of Zurich and University Hospital Zurich Zurich Switzerland; ^5^ Department of Health Sciences, Translational Cardiovascular Technologies ETH Zürich Zürich Switzerland; ^6^ Department of Cardiothoracic and Vascular Surgery German Heart Institute Berlin Berlin Germany; ^7^ Department of Trauma and Orthopedic Surgery University Hospital Erlangen, Friedrich‐Alexander‐University Nürnberg Erlangen Germany

**Keywords:** femur fracture, fracture hematoma, immune cell homeostasis, intramedullary nailing, reaming, RIA

## Abstract

The local inflammatory impact of different reaming protocols in intramedullary nailing has been sparsely investigated. We examined the effect of different reaming protocols on fracture hematoma (FH) immunological characteristics in pigs. To do so, a standardized midshaft femur fracture was induced in adult male pigs. Fractures were treated with conventional reamed femoral nailing (group RFN, *n* = 6); unreamed femoral nailing (group UFN, *n* = 6); reaming with a Reamer Irrigator Aspirator device (group RIA, *n* = 12). Animals were observed for 6 h and FH was collected. FH‐cell apoptosis and neutrophil receptor expression (Mac‐1/CD11b and Fc*γ*RIII/CD16) were studied by flow cytometry and local temperature changes were analyzed. The study demonstrates that apoptosis‐rates of FH‐immune cells were significantly lower in group RIA (3.50 ± 0.53%) when compared with non‐RIA groups: (group UFN 12.50 ± 5.22%, *p* = 0.028 UFN vs. RIA), (group RFN 13.30 ± 3.18%, *p* < 0.001, RFN vs. RIA). Further, RIA‐FH showed lower neutrophil CD11b/CD16 expression when compared with RFN (mean difference of 43.0% median fluorescence intensity (MFI), *p* = 0.02; and mean difference of 35.3% MFI, *p* = 0.04, respectively). Finally, RIA induced a transient local hypothermia and hypothermia negatively correlated with both FH‐immune cell apoptosis and neutrophil activation. In conclusion, immunologic changes observed in FH appear to be modified by certain reaming techniques. Irrigation during reaming was associated with transient local hypothermia, decreased apoptosis, and reduced neutrophil activation. Further study is warranted to examine whether the rinsing effect of RIA, specific tissue removal by reaming, or thermal effects predominantly determine local inflammatory changes during reaming.

## 1 INTRODUCTION

The constitution and quality of fracture hematoma (FH) play an important role in fracture healing. Removal of FH or repeated early FH debridement has shown deleterious effects on fracture healing.^1^, ^2^ Intramedullary reaming can also modify FH, most likely due to changes in intramedullary blood flow, intramedullary pressure, and intramedullary temperature.^3^, ^4^, ^5^, ^6^ Kinetics of humoral factors caused by changes in FH have been studied before. Likewise, the importance of both local and systemic cellular factors on bone repair has previously been explored.^7^, ^8^, ^9^, ^10^, ^11^ After injury, circulating immune cells promptly infiltrate the rapidly changing FH and contribute to the regulation of local growth and differentiation factors, which have been shown to regulate the fracture healing process. Within hours, polymorphonuclear neutrophils (PMNs) within the FH outnumber all other immune cells.^7^, ^8^, ^9^ Some authors have correlated an excessive influx of activated neutrophils into FH with compromised fracture healing.^11^

Conventional reaming versus application of the Reamer Irrigator Aspirator (RIA) has been linked with more sustained systemic inflammatory changes.^6^ Further, an association between inflammatory changes and systemic temperature alterations early after fracture was shown.^12^, ^13^ Finally, our group has previously demonstrated that RIA is associated with less fat intravasation, decreased systemic immunologic changes, and reduced sequelae in concomitant acute chest trauma.^14^, ^15^, ^16^ However, it is unclear whether the irrigating effects may also have a measurable influence on local inflammation or immune cell composition.

Our group has previously used a standardized large animal model to (1) assess changes in the circulating immune response,^12^ (2) create a standardized mid‐shaft femoral fracture,^17^ (3) modulate immunologic changes by inducing hypothermia,^12^, ^13^ and (4) measure regional changes in circulation and inflammation.^18^

We utilized this standardized porcine femur fracture model to test the following hypotheses with respect to direct local effects of RIA on FH in addition to other known changes induced by RIA:

i. Application of rinsing techniques (RIA) is associated with regional immunological changes in fracture hematoma.
ii. Among other known changes induced by RIA (blood flow, removal of tissue) the effect on local temperature changes and associated immune cell responses can be effective.

## 2 METHODS

The current study was approved by the local Official Veterinary Office (Kanton Zürich, Gesundheitsdirektion Veterinäramt under Project number: ZH138/17). All animal experiments were designed and carried out in accordance with the “Guide for Care and Use of Laboratory Animals.”^19^ Data processing and documentation have been performed in line with the ARRIVE guidelines for reporting animal research.^20^

### 2.1 Experimental model and study groups

A standardized animal protocol was applied, as previously described.^10^, ^12^, ^17^ Briefly, experiments were performed using 24 male Swiss Large White pigs (4 months old animals weighing 50 ± 5 kg). Before the start of the experiment, animals received pre‐medication by intramuscular injection of ketamine (Ketasol®‐100, Dr. E. Graeub AG) 15 mg/kg, midazolam (Dormicum®, Roche Pharma [Schweiz] AG) 0.5 mg/kg, and atropine (Atropin 1%, Kantonsapotheke Zurich) 0.05 mg/kg. Then, general anesthesia was induced and maintained with a mixture of propofol (Propofol® Lipuro, B. Braun Medical AG; 5–10 mg/kg/h CRI) and sufentanil forte (Sufenta® Forte, Janssen‐Cilag AG; 0.01 mg/kg/h CRI). After intubation (Bivona®, ID: 9 mm, OD: 12.4 mm, 37FR, Length: 56 cm, Balloon: 5 cm), a lung protective volume‐controlled ventilation regime was applied. Ventilation settings were frequently optimized based on routine blood gas analysis and capnometry to target a pCO_2_ of 35–45 mmHg. Percutaneous placement of an arterial line, two‐lumen central venous catheter (HighFlow Dolphin Catheter, 13F, Baxter International), and a suprapubic catheter were performed after intubation.

Crystalloids (Ringerfundin 2 ml/kg BW/h) were administered continuously and hemodynamic and metabolic parameters were assessed at set time points.

Then, all animals underwent standardized unilateral femoral fracture as previously described.^10^, ^12^, ^17^ Briefly, a bolt gun machine (Blitz‐Kerner, turbocut JOBB GmbH) and a custom‐made metal plate were applied to produce a standardized midshaft transverse femur fracture. Fracture location and morphology were confirmed by fluoroscopy.

In the first 90 min after trauma, the preclinical phase was mimicked with altered ventilator settings, no temperature correction, reduced fluid infusion of 10 ml/h, and FiO_2_ reduction to ambient air oxygen level of 0.21.

After circulatory stabilization, all 24 animals were randomly assigned to one of the following three study conditions:

i. unreamed femoral nailing/(group UFN, *n* = 6)
ii. reamed femoral nailing/(group RFN, *n* = 6), or
iii. Reamer Irrigator Aspirator enhanced RFN/(group RIA, *n* = 12).

### 2.2 Surgical intervention

**Figure 1:**
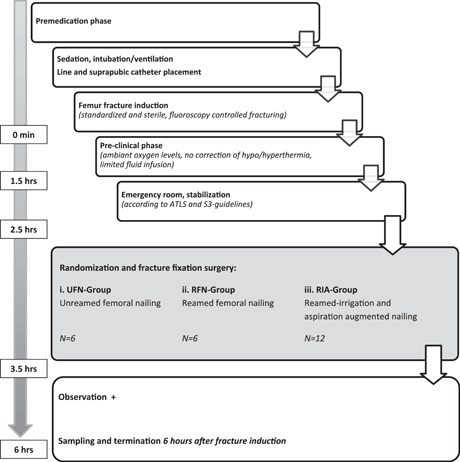
This figure illustrates the experimental design of the standardized study and the flowchart summarizes all phases of the experiment

The standardized nailing system utilized a tailored 120 mm nail (cannulated DFN Ø 10.0 mm, DePuy Synthes). In animals randomized for RFN, standardized stepwise intramedullary reaming preceded nailing. The SynReam intramedullary reaming system (DePuy Synthes) was utilized and reamer heads with a diameter up to 12 mm were used. In the RIA group, a Synthes Reamer/Irrigator/Aspirator System (DePuy Synthes) was used according to protocol. Again, reamer heads of 12 mm were utilized. Reamer heads were replaced after every five experiments in all study groups. Sequential reaming at intervals of 0.5 mm was performed. Final reaming with the 12 mm drill head was repeated twice. To optimize standardization of our study conditions, all fractures treated by a treatment modality including intramedullary nailing, have been reamed 10 times in total. The experimental design is presented as a flowchart in Figure 1.

**Figure 2:**
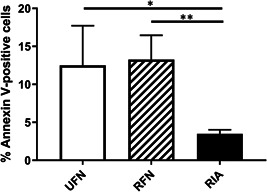
This figure displays the impact of reaming strategy on early immune cell apoptosis in fracture hematoma. Immune cell hypothesis was determined 6 h after insult. Bars represent the percentage (mean ± *SEM*) of Annexin‐V‐positive immune cells following different reaming protocols. *p < 0.05; **p < 0.01

### 2.3 Systemic body temperature regulation

Core temperature (*T*_core_) was monitored by using an esophageal temperature probe connected to a Datascope Passport2 Patient Monitoring System (Pacific Medical). Core temperature was corrected by: (1) controlling room temperature, (2) administering pre‐heated fluid infusions, and (3) warming blanket adjustment (Bair Hugger, 3M).

A local temperature probe (Electronic Precision Thermometer ama‐digit ad 15th, Amarell GmbH & Co. KG) was placed near the fracture site (*T*_tissue_). Standardized probe positioning was achieved by fluoroscopically guiding the probe one shaft diameter lateral to thefracture. . During surgery, temperature was measured continuously, and peak values (lowest and highest intraoperative temperatures) were compared between groups.

### 2.4 Termination and tissue collection

Animals were terminated after an observation period of 6 h with a Na‐pentobarbital (Esconarkon ad us. Vet., Streuli Pharma AG) infusion. Before termination, samples of FH were collected.

### 2.5 Laboratory analysis and flow cytometry

FH‐single cell solutions were made by flushing/crushing collected FH‐material. Thereafter, FH samples were continuously kept on ice. The solution was centrifuged for 4 min at 1500 rpm at 4°C, and supernatants were taken up in RBC‐lysing buffer (BioLegend). After lysis, FACS‐buffer (phosphate‐buffered saline [PBS] enriched with 0.5% bovine serum albumin and 0.5 mM EDTA) was added to the samples and two washing steps (4 min, 1500 rpm at 4°C) were performed. Thereafter, conjugated antibody mixes were added and allowed to incubate for 45 min. The following antibodies were utilized: CD11b (Clone 2F/11, AbdSerotec), CD16 (Clone G7, AbdSerotec), and CD45 (Clone K252.1E4, AbdSerotec). After incubation, two additional washing steps were performed and solutions were directly analyzed by flow cytometry. A standardized Annexin‐V staining protocol was utilized to test for cell apoptosis (Abcam). A Canto II‐device (Becton & Dickinson) and FACS Diva Software (Becton & Dickinson) were used 1,2.

CD11b (Mac‐1) is an integrin family member. Functionally CD11b regulates neutrophil adhesion and migration. Moreover, CD11b is a traditional neutrophil‐activation marker and cell activation is characterized by upregulation of this integrin in both in vitro and in vivo studies.^21^, ^22^, ^23^

CD16 (Fc*γ*RIII) is an important and well‐described Fc*γ*‐receptor. This receptor binds to immunoglobulins (IgG) either in aggregates or attached to pathogens. Binding of IgG to Fc*γ* receptors promotes the oxidative burst and induces phagocytosis.^24^ Short term in vitro activation of neutrophils by lipopolysaccharide is associated with an initial increase in neutrophil CD16‐expression.^25^ Neutrophil Fc*γ*RIII‐receptor expression increases during cell maturation, with young neutrophils typically having relatively low CD16‐cell surface expression compared to older counterparts.^26^, ^27^

CD45 is a pan‐leukocyte marker and is utilized to identify immune cells in blood and the tissue compartment.^28^

FlowJo (Becton & Dickinson) was utilized for the evaluation of flow cytometry data and the gating strategy utilized is summarized in Supporting Information 1. Leukocyte subtype identification is based on forward‐sideward scatter gating and CD45‐expression levels. This protocol has previously been validated in pilot experiments using both porcine blood and hematoma samples. Differential cell counts were obtained with cytospin‐prepared slides stained with May–Grünwald–Giemsa.

### 2.6 Sample size calculation

Sample size calculations were based on tissue neutrophil presence after intervention. In a previous study on pulmonary neutrophil migration after polytrauma performed by this study group, 12 animals were exposed to polytrauma while 6 animals were included in a sham control group. Neutrophil presence in lung tissue in the intervention group (*n* = 12) was determined as 2.38 (*SD*: 0.76) cells per high power field, whereas 0.98 (*SD*: 0.2) cells/high power field were counted in the sham group (*n* = 6). This results in power of 98% if the sample size in two groups is 4 at a 5% significance level.^29^ However, the number of animals and the allocation ratio of 2:1:1 in the current study were based primarily on logistic and ethical considerations instead of a formal a‐priori sample size calculation. Sample sizes of 12 and 6 in the groups were used to avoid an underpowered study protocol.

### 2.7 Statistical analysis

All statistical analyses were performed using GraphPad Prism Version 7. Unpaired *t*‐tests (normally distributed datasets) or Mann–Whitney *U*‐tests (nonparametrical analysis) were performed. Correlation analysis and plotting were performed with Excel (Microsoft) and a web‐based Pearson Correlation Coefficient Calculator, retrieved from Social Science Statistics at www.socscistatistics.com. A *p *value < 0.05 was considered statistically significant. All data are presented as standard error of the mean (SEM).

## 3 RESULTS

All 24 animals survived the observation period. No intra‐ or postoperative complications were observed.

### 3.1 FH‐immune cell subpopulation composition

Flow analysis demonstrated no differences in FH‐white blood cell subpopulation composition between the different study groups (Supporting Information 2). Overall, the majority (63.12 ± 2.72%) of isolated FH leukocytes (defined as all nucleated/CD45^+^‐cells) were identified as granulocytes. Additional morphological analysis demonstrated that among the granulocytes, PMNs were the most prevalent FH‐immune cell type. More specifically, 61.23 ± 2.18% of FH‐white blood cells were identified as PMNs. The second most predominating type of leukocyte in FH was lymphocytes (32.01 ± 2.75% of all nucleated CD45^+^‐cells). Finally, 1.85 ± 0.37% of FH‐immune cells showed morphology characteristic of eosinophils, while 4.21 ± 0.98% of cells were monocytes/macrophages and less than 1% of FH‐immune cells were basophils.

### 3.2 FH‐immune cell apoptosis rates

FH‐immune cell apoptosis rates were significantly lower in the RIA group (3.50 ± 0.53%/ Annexin‐V^+^/FH−CD45^+^cells) than in the UFN and RFN groups (12.50 ± 5.22% Annexin‐V^+^/FH‐CD45^+^ cells, *p* = 0.028 and 13.30 ± 3.18% Annexin‐V^+^/FH‐CD45^+^ cells, *p* < 0.001, respectively). Of note, immune cell apoptosis rates did not differ between the UFN and RFN groups (*p* = 0.90). Apoptosis rates are displayed in Figure 2.

### 3.3 Neutrophil Mac‐1 (CD‐11b) and Fc*γ*RIII‐receptor (CD‐16) expression profiles of FH‐PMNs

**Figure 3:**
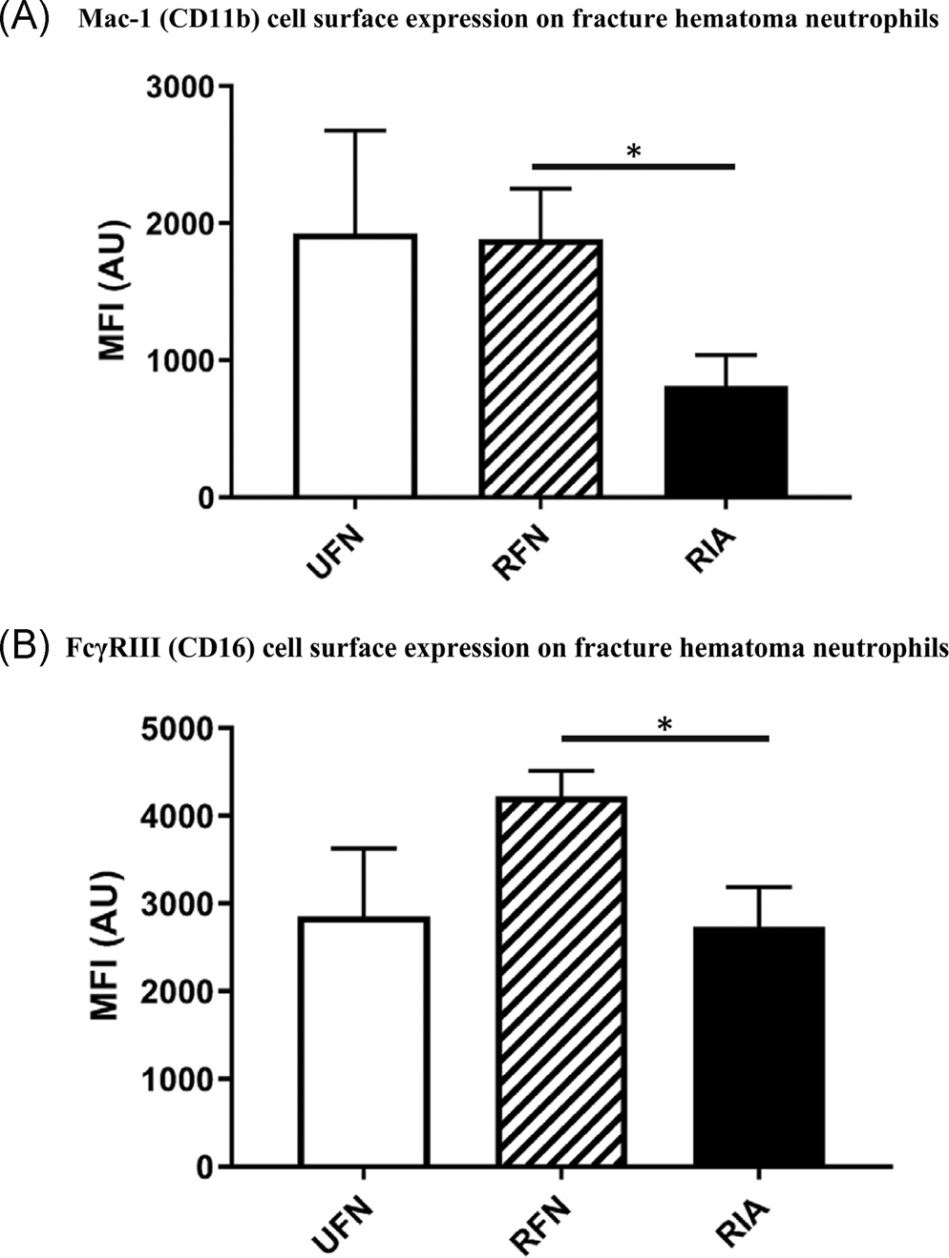
(A) This figure displays differences in neutrophil Mac‐1 cell/CD11b surface expression on fracture hematoma (FH) neutrophils following different reaming protocols. Bars represent the median fluorescent intensity (MFI) in arbitrary units (AUs) of Mac‐1 on neutrophils in FH determined by flow analysis per study conditions. Study groups have been compared. *, *p* < 0.05. (B) This figure displays differences in neutrophil Fc*y*RIII cell/CD16 surface expression on FH neutrophils following different reaming protocols. Bars represent the MFI in AUs of Mac‐1 on neutrophils in FH determined by flow analysis per study conditions. Study groups have been compared. *p < 0.05

Mac‐1 cell surface expression levels on FH‐PMNs were significantly lower in the RIA group compared with the RFN group (*p* = 0.02). In addition, Fc*y*RIII‐expression in FH‐PMNs was also significantly lower in the RIA group compared to the RFN group (*p* = 0.04). FH‐PMN cell surface receptor expression levels observed in the different study groups are summarized in Figure 3.

### 3.4 In vivo fracture site temperatures

With comparable baseline temperature levels, fracture site temperatures during surgery were lower in the RIA‐group (mean *T*_tissue/min_: 34.5 ± 0.3°C) versus the UFN group (35.7 ± 0.2°C, *p* = 0.0194) and the RFN group (36.1 ± 0.3°C, *p* = 0.002. A representative example of temperature probe positioning is shown in Supporting Information 3.

Higher intraoperative mean peak temperatures were encountered in the RFN group (37.2 ± 0.4°C) versus the UFN group (35.8 ± 0.2°C, *p* = 0.0194) and RIA‐reamed group (35.3 ± 0.397°C, *p* = 0.0079). Local temperature changes are shown in Table 1.

**Figure 4:**
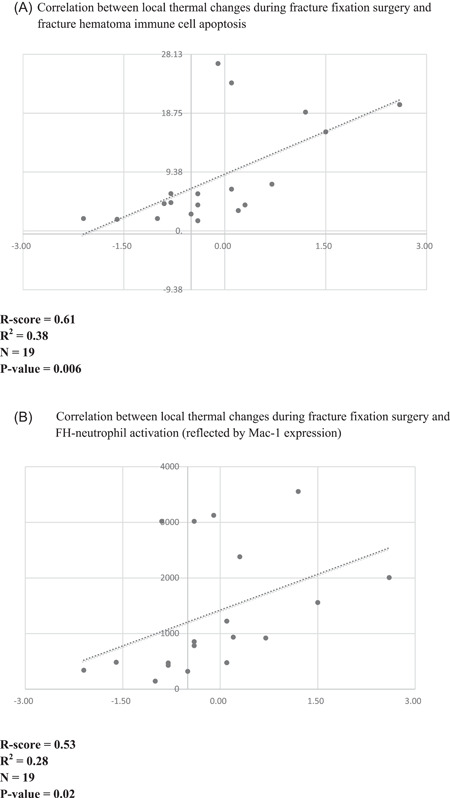
(A) This figure demonstrates the correlation between local thermal changes during fracture fixation surgery and fracture hematoma (FH) immune cell apoptosis. *y‐axis*: percentage of early FH‐immune cell apoptosis in FH as measured by percentage of Annexin‐V positive immune cells; *x‐axis*: temperature coefficient (in °C). All dots represent individual samples. (B) This figure demonstrates the correlation between local thermal changes during fracture fixation surgery and FH neutrophil activation. FH‐neutrophil activation is determined by neutrophil CD11b cell surface expression levels at termination. *y‐axis*: neutrophil CD11b/Mac‐1 expression in early FH; *x‐axis*: temperature coefficient (in °C). All dots represent individual samples

As shown in Figure 4, intraoperative temperature and early FH‐immune cell apoptosis were strongly correlated (*r*(17) = 0.61, *p* = 0.006), while neutrophil activation in early FH moderately correlated with local tissue temperature (*r*(17) = 0.53, *p* = 0.02).

**Table 1:** Differences in local temperature alterations during various fracture fixation strategies

Condition:	T_core_ :baseline	T_tissue_ :baseline	T_tissue_ :intra‐operative min	T_tissue_ :intra‐operative max	T_coefficient_ :intra‐operative
*UFN*	37.4	35.8	35.7	35.8	+0.020
	(SEM 0.230)	(SEM 0.181)	(SEM 0.227)§	(SEM 0.150)**	(SEM 0.073)§, **
*RFN*	37.0	36.1	36.1	37.2	+1.15
	(SEM 0.193)	(SEM 0.270)	(SEM 0.262)*	(SEM 0.428)*, **	(SEM 0.339)*, **
*RIA‐nailing*	37.4	35.2	34.5	35.3	‐0.890
	(SEM 0.165)	(SEM 0.083)	(SEM 0.282)^§,^ *	(SEM 0.397)*	(SEM 0.179)^§,^ *

Differences in core temperature at baseline (T_core_), local tissue temperature at the fracture site prior to surgery (T_tissue_), lowest tissue temperature during fracture fixation (T_tissue/min_), peak tissue temperature during fracture fixation (T_tissue/max_) and the temperature coefficient (T_coefficient_).

^§^ P<0.05 RIA‐enhanced reaming vs. UFN

* P<0.05 RIA‐enhanced reaming vs. RFN

** P<0.05 UFN vs. RFN

## 4 DISCUSSION

Studies have shown that fracture‐healing success is driven largely by the early inflammatory stages after injury. This inflammatory phase sets the stage for the second phase of fracture healing, the repair process.^1^, ^2^, ^30^ Hauser et al.^7^ have demonstrated that 4‐h FH is rich in activated monocytes, neutrophils, and soluble cytokines such as interleukin 6/8 and 10. In contrast to the local humoral immune response at the fracture site,^7^, ^8^, ^10^ the regional cellular immune response is still poorly understood. The current study is the first to describe fundamental characteristics of FH‐immune cell content. Furthermore, this study describes the impact of different reaming strategies on FH‐immune cell homeostasis and their potential interplay with local temperature changes during surgery.

Excessive systemic immune activation precedes inadequate bone healing by altering the immunological composition of early FH.^31^, ^32^, ^33^, ^34^ Specifically, studies suggest that excessive influx of activated neutrophils into FH is associated with inadequate fracture healing.^11^ Despite varying reaming strategies, PMN levels in FH were consistent across all groups in the current study. Interestingly, however, while no quantitative differences in immune cell composition were found, prominent variations in FH neutrophil activation status were encountered between groups.

Based on this revelation, the key findings in this study may provide relevant new information:

1. Neutrophils were the most prevalent FH‐immune cells and the composition of immune cell subtypes was unaffected by reaming strategy in nail fixation.
2. RIA was associated with diminished FH‐immune cell apoptosis compared with UFN and RFN. Further, RIA was associated with lower FH‐neutrophil activation compared with RFN.
3. RIA induced local hypothermia at the fracture site, and negatively correlated with both FH‐immune cell apoptosis and neutrophil pool activation.

Sheep experiments have shown that leukocyte counts in early FH start to rise immediately after fracture induction. The percentage of FH‐myeloid cells also increases immediately after injury, with FH concentrations equaling circulatory levels within 4 h. During this time period, however, the proportion of nonviable leukocytes in FH also increases.^8^, ^9^, ^10^ The current investigation is the first to demonstrate that *fracture fixation strategy*, or more specifically, reaming strategy, influences white blood cell apoptosis in FH. Specifically, reamed‐irrigation augmented nailing is associated with less early cell apoptosis compared with unreamed and stepwise reaming protocols.

The current study demonstrated, in line with other animal^7^ and human trauma studies,^35^ that neutrophils are initially the most prevalent immune cells in early FH. This study, further reveals that the percentage of myeloid cells (including neutrophils) in 6‐h FH was not affected by reaming strategy. Interestingly, however, RIA‐enhanced reaming was associated with diminished FH neutrophil activation, demonstrated by lower levels of PMN‐CD11b surface expression,^21^, ^36^ compared with the other study groups. It is tempting to hypothesize that irrigation/aspiration induces less profound regional immune cell activation than alternative reaming methods. Knowledge about which specific characteristics of the RIA technique modulate early FH‐immune cell response is important as it could guide the development of future reaming strategies.

Since this study demonstrated a correlation between in vivo local temperature and local neutrophil activation, it is tempting to speculate that observed differences in PMN‐activation levels between the study groups may have primarily been a function of thermal differences between the reaming strategies. These findings are in line with in vitro studies that assessed neutrophil activation in the field of pediatric extracorporeal circuits and cardiac bypass surgery in which cooling procedures decreased neutrophil CD11b expression.^37^ Furthermore, in vitro experiments evaluating different neutrophil preservation strategies, showed that PMNs at 37°C versus 4°C exhibited a 2.5‐fold increase in formyl‐methionyl‐leucyl‐phenylalanine (FMLP)‐receptor expression. In addition, increased cellular functional activity, demonstrated by increased levels of FMLP‐induced superoxide generation, was seen as well.^38^ Human PMNs heated to temperatures above 37°C exhibited higher levels of reactive oxygen intermediates release upon in vitro lipopolysaccharide stimulation compared with PMNs stored at lower temperatures.^39^

The importance of thermal regulation on local immune cell homeostasis has previously been posited by our group based on data from a standardized hypothermia model in the setting of porcine polytrauma that was associated with reduced hepatic granulocyte infiltration compared with normothermia.^13^ However, experimentally induced hypothermia in a long‐term porcine model of combined trauma led to prolonged (48 h) enhancement of systemic and remote humoral parameters (including high mobility group box 1 and interleukin‐6) compared with normothermic controls.^12^

A correlation between thermal changes and FH‐immune cell apoptosis was also observed. It has previously been demonstrated that relatively moderate temperature changes within febrile temperature ranges trigger neutrophilic cell apoptosis. Within just fifteen minutes of culturing neutrophils at 39.5°C, Caspase‐8 reaches peak levels. Interestingly, Caspase‐8 inhibition protects cells from heat‐induced apoptosis.^40^ Further, in vitro studies have demonstrated that mild hypothermia protects mammalian cells from apoptosis induced by various stimuli.^41^

Reamer head design is an important factor in local heat production during reaming. Utilization of a large and blunt reamer has been shown to exacerbate local temperature elevation.^42^, ^43^ Given the increased local temperatures observed during conventional reaming in this study, temperature likely contributed to increased FH‐immune cell apoptosis in the RFN conventional reaming group, while RIA‐induced transient local hypothermia induced immune cell preservation.

Further, conventional RFN was associated with enhanced FH‐neutrophil CD16‐receptor expression, which is considered an essential receptor in the phagocytic process.^44^ Decreased PMN‐CD16‐expression after RIA may be due to the influx of immature band neutrophils from the bone marrow.^45^, ^46^ Further deciphering CD16 expression in long bone reaming is certainly an area of future study. Additionally, future studies should more specifically investigate the impact of local hypothermia on FH neutrophil homeostasis, for example, through varying cooling protocols during reaming.

These study results must, however, be qualified considering several possible limitations. First, this experiment focused on the early cellular immune reaction at the fracture site, which is dominated by neutrophils. Long‐term effects of different reaming protocols were therefore not investigated. Furthermore, to avoid artificial manipulation at the fracture site, local temperature probes were not inserted directly into the fracture but were fluoroscopically guided to a standardized position near the fracture site. As a result, FH temperature measurements in this study were indirect, but consistent approximations across all study groups. Further, as humoral immune characteristics of FH have been studied in detail before,^1^, ^7^, ^10^, ^12^ we decided to focus on immune effector cells only. Therefore, we were unable to determine the interplay between early humoral and cellular immune response in FH. For the current large‐animal study, 4‐month‐old male animals were used. Four‐month‐old pigs are considered young adults. These animals at this age, therefore, represent the most common patient group with transverse mid‐shaft femur fractures. To avoid the potential confounding effect of hormonal factors, male animals were chosen. Therefore, caution should be taken in extrapolating our experimental findings to other trauma subgroups (e.g., women/geriatric patients).

In conclusion, this standardized porcine study appears to suggest that RIA‐induced transient local hypothermia in bone and soft tissues may reduce early immune cell apoptosis and decrease neutrophil activation in FH. The current study further revealed that FH‐immune cell composition is not affected by reaming strategy. These findings inspire further investigation and should serve as a foundation for designing new, enhanced reaming protocols that may ultimately help optimize fracture healing.

## CONFLICTS OF INTEREST

The authors declare no conflicts of interest.

## AUTHOR CONTRIBUTIONS

Michel P. J. Teuben, Sascha Halvachizadeh, Yannik Kalbas, Zhi Qiao, Nikola Cesarovic, Miriam Weisskopf, Miriam Kalbitz, Paolo Cinelli, and Roman Pfeifer performed the experiments including animal studies and laboratory studies. Michel P. J. Teuben, Sascha Halvachizadeh, Henrik Teuber, Miriam Kalbitz, Roman Pfeifer, and Hans‐Christoph Pape wrote the paper. Michel P. J. Teuben, Roman Pfeifer, Paolo Cinelliand Hans−Christoph Pape contributed to the experimental design and coordinated the study, and supervised financial support for the studies. Michel P. J. Teuben was the principal investigator of the project. All authors made substantial contributions to the conception and design of the study, participated in drafting the article, and gave final approval of the version to be published.

## Supporting information

Supporting information.
