# Supplementary material for: Cellular activation status in femoral shaft fracture hematoma following different reaming techniques – A large animal model
Source: J Orthop Res. 2022 Mar 17;40(12):2822–30. doi: 10.1002/jor.25309 (PMC9790649; doi:10.1002/jor.25309)

**Supplement 1.** Multistep gating strategy of porcine fracture hematoma leukocyte subtypes

1.
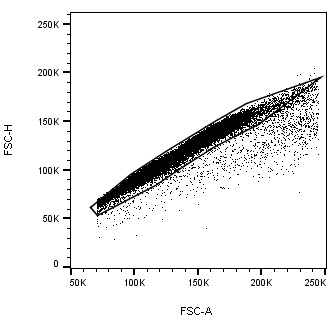
**Singlet gating: *exclusion of doublets***
2.
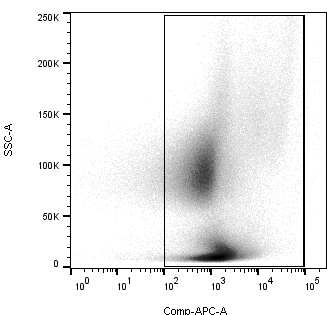
**CD45+/SSC-gating of FH-immune cells: *purification of immune cells***
3.
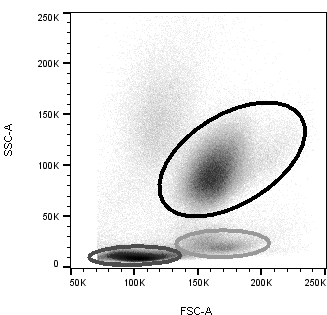
**FSC/SSC-gating of specific leukocyte subpopulations: *leukocyte subtype identification***

**Granulocytes: neutrophils/CD16+
eosinophils/CD16-**

**Monocytes/macrophages**

**Lymphocytes**

**Supplement 2:** Early fracture hematoma white blood cell content determined by morphological analysis of cytological samples

**
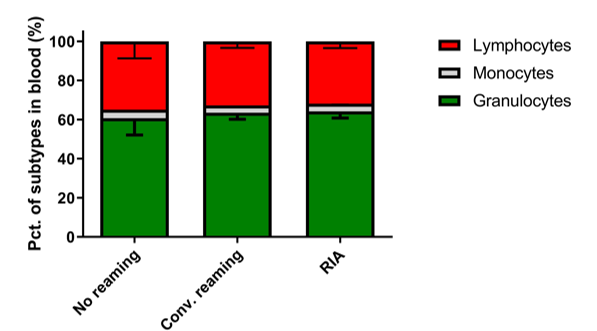
**

**UFN**

**RFN**

**RIA**

Supplement 2. **Granulocytes were the most prominent immune cell type in early fracture hematoma. Furthermore, the immune cell composition of fracture hematoma was not affected by reaming protocol.**

**Supplement 3.** Representative example of fluorescence-guided standardized temperature probe positioning


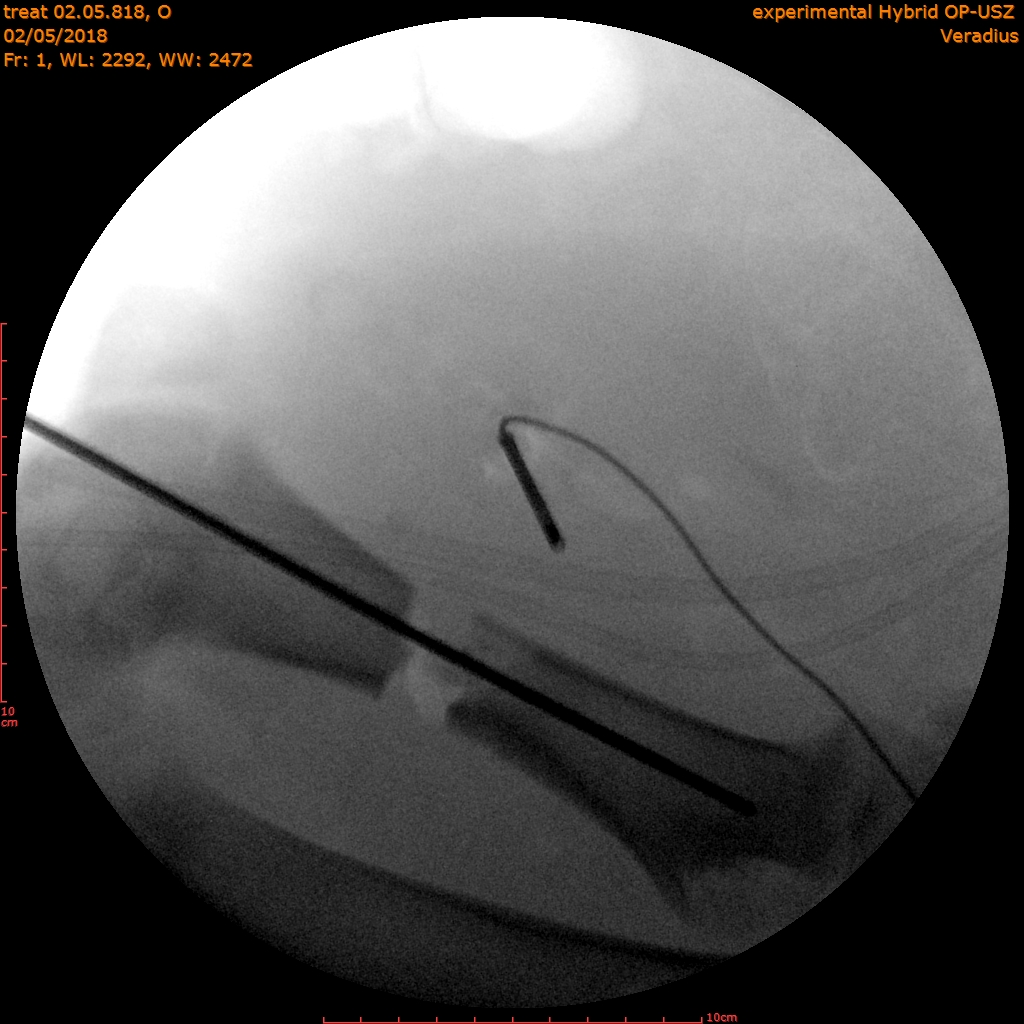

Supplement: Supplementary file 1 — Supporting information. [file JOR-40-2822-s001.docx]
